# Intra-articular Hyaluronic Acid (HA) and Platelet Rich Plasma (PRP) injection versus Hyaluronic acid (HA) injection alone in Patients with Grade III and IV Knee Osteoarthritis (OA): A Retrospective Study on Functional Outcome

**DOI:** 10.5704/MOJ.1607.007

**Published:** 2016-07

**Authors:** C Saturveithan, G Premganesh, S Fakhrizzaki, M Mahathir, K Karuna, K Rauf, H William, H Akmal, N Sivapathasundaram, K Jaspreet

**Affiliations:** Department of Orthopaedics, Malacca General Hospital, Malaysia; * Department of Radiology, Malacca General Hospital, Malaysia

**Keywords:** Hyaluronic Acid and Platelet Rich Plasma, Grade III and IV Knee Osteoarthritis

## Abstract

**Introduction:** Intra-articular hyaluronic acid (HA) is widely utilized in the treatment of knee osteoarthritis whereas platelet rich plasma (PRP) enhances the regeneration of articular cartilage. This study analyses the efficacy of HA and PRP in grade III and IV knee osteoarthritis.

**Methodology:** This is a cross sectional study with retrospective review of 64 patients (101 knees) which includes 56 knees injected with HA+ PRP, and 45 knees with HA only.

**Results:** During the post six months International Knee Documentation Committee (IKDC) evaluation, HA+PRP group showed marked improvement of 24.33 compared to 12.15 in HA group. Decrement in visual analogue score (VAS) in HA+PRP was 1.9 compared to 0.8 in HA group.

**Conclusion:** We propose intra-articular HA and PRP injections as an optional treatment modality in Grade III and IV knee osteoarthritis in terms of functional outcome and pain control for up to six months when arthroplasty is not an option.

## Introduction

Knee osteoarthritis (OA) is the most common form of chronic arthritis causing severe pain, disability, loss of function and affecting the quality of life of the patients^1^.

Studies have shown that 15% of the world population suffers from osteoarthritis which includes 39 million people in the European countries and more than 20 million of Americans^2^. The number of patients affected is on the rise and by 2020 this figure would have probably multiplied^2^. In Malaysia, 9.3% of adult Malaysians have knee pain and more than half of them have clinical evidence of OA. The prevalence ranges from 1.1% to 5.6% in the various ethnic groups in Malaysia^3^.

Knee OA is characterized by degeneration of the articular cartilage which will eventually lead to the joint destruction^4,5^. The underlying causes of OA are multifactorial with several predisposing factors such as mechanical trauma, obesity, genetic factors, inflammatory joint disease, previous joint infection, advancing age, metabolic factors, osteoporosis, and ligamentous laxity^6^. Diagnosis of OA is made with clinical assessment and radiological investigation as an adjunct^7,8^. Less than 50% of patients with radiological changes of osteoarthritis are symptomatic; therefore, treatment is based on symptoms rather than radiological changes^9^.

The mainstay of treatments for early stage of knee osteoarthritis are analgesics, activity modification and physiotherapy. Over time, patients usually become refractory to the initial treatment regime, hence reconstructive surgery becomes the subsequent treatment modality. Analgesics widely used in knee OA patients only help in reducing inflammation and pain but they are ineffective in delaying disease progression^10^. Currently, there are numerous ongoing efforts to develop new tissue engineering- based strategies for treatment of OA^11^. Recent studies show that drugs such as glucosamine, chondroitin sulphate and intra-articular injections of hyaluronic acid not only favor pain relief, but also prevent the progression of the disease^12,13^.

Hyaluronic acid (HA) is a naturally occurring polysaccharide in the synovial fluid and is responsible for the elastoviscocity of synovial fluid^13^. It is a large glycosaminoglycan which is composed of long repeating disaccharides of glucuronic acid and N-acetylglucosamine^14,15^. Viscosupplemention with intraarticular HA in knee osteoarthritis is widely regarded as an effective treatment in improving pain and function due to its protective effect on articular cartilage, acting as lubricant and shock absorbent^16,17^.

Contemporary studies in the developed countries have demonstrated the additional use of platelet rich plasma (PRP) in treatment of knee osteoarthritis. PRP is platelet concentrate (2-10 times of baseline concentration) that is obtained from patient’s autologous blood sample by centrifugation^18^. Platelets store more than 1500 active proteins in α and dense granules^19,20^. α granules contain numerous growth factors (GF) like platelet derived GF(PGDF), transforming GF(TGF-β), platelet-derived epidermal GF, vascular endothelial GF, insulin like GF-1, fibroblastic GF and epidermal GF which promotes healing potential in degenerating articular cartilage^21,22^. Meanwhile, the dense granules contain adenosine diphosphate, adenosine triphosphate, calcium, histamine, serotonin, and dopamine which aids in regeneration of the degenerated tissues^20^. Studies have suggested that the combination of HA and plasma rich with growth factors enhance the regeneration of articular cartilage^23^.

The objective of this study is to analyse the efficacy of intraarticular injection of HA and PRP versus HA injection alone in patients with grade III and IV knee osteoarthritis patients. It is envisaged that this study will make a significant contribution in improving the quality of life in patients suffering from severe knee osteoarthritis who are not fit for operation, not keen for surgical intervention or financially restricted.

## Methodology

This is a cross-sectional study with retrospective review of medical records which was conducted from October 2013 to April 2014 at Malacca General Hospital and has been approved by the hospital and National Clinical Research Centre (CRC) Ethics Committee. Data was collected and reviewed from intra-articular knee injections registry. A total of 254 patients had received intra-articular knee injection from October 2013 to March 2014. Of the 254 patients, 70 had grade III or grade IV knee osteoarthritis.

Our inclusion criteria was patients with Grade III and IV primary knee OA based on Kellgren Lawrence classification, who received 4 ml High Molecular Weight Hyaluronic Acid (HMWHA) with concentration of 22mg/ml alone or with combination of PRP. This is due to the availability of multiple concentrations of hyaluronic acid in practice which may cause inconsistency of the final results.

Patients with grade I and II knee osteoarthritis, secondary knee osteoarthritis, patients who received HA injection with concentration and molecular weight other than mentioned in inclusion criteria, patients with osteoarthritis of other joints, those with other inflammatory and non-inflammatory joint disease, coagulopathies and with local or systemic infections were excluded from this study.

Sixty-four patients (101 knees) who fulfilled the criteria were selected and the six patients were excluded based on the exclusion criteria. The study group comprised 24 men and 40 women, with an average age of 66 (range 50 - 87) years. One hundred and one knees had received intraarticular injection; 37 patients with bilateral knee injections (74 knees) and 27 patients with unilateral knee injection. Of these, 56 knees had received HA and autologous PRP combination intra-articular injections, whereas 45 knees had received only HA injections.

Knee assessment and pain score were routinely recorded and documented in our centre with International Knee Documentation Committee (IKDC) questionnaires and Visual Analogue Scale (VAS) prior to their injections, and after two months and six months following injection. IKDC was chosen over other measurement tools like Knee Injury and Osteoarthritis Outcome Score (KOOS), and Western Ontario and McMaster Universities Arthritis Index (WOMAC) due to its high reliability and validity. Higgins *et al* have concluded that it is the ideal measurement tool to assess functional outcome as it showed the best performance on all measurement properties^24^.

The data were collected and reviewed from the medical records of the selected patients. The retrospectively collected data were then analysed using SPSS version 18 with paired t-test. The statistical analysis was conducted with 95 percent confidence interval and a p-value of <0.05 as threshold of statistical significance.

We have standardized patient selection by only including patients who had received specific HA and PRP concentration or preparation method. All patients included in the study had received 4 ml high molecular weight (1.476 x 10^6^ average Daltons) hyaluronic acid with concentration of 22mg/ml. HMW HA was specifically selected because many earlier studies showed greater and longer efficacy in reducing pain and other symptoms and recovering articular function as compared to LMW HA^25, 26^.

The PRP injections were standardized among all our patients as the PRP preparation is done routinely in our centre with a standard operating procedure. It is prepared by withdrawing 30 ml of patient’s own venous blood, added with anticoagulant, and - centrifuged by duo-spin method, at the rate of 2500 rpm for first 5 minutes and then 3200 rpm for the next 10 minutes (total of 15 minutes). Three separate layers are produced at the end of the centrifugation: plasma, buffy coat (platelet) and RBCs. Ninety-five percent of the plasma layer is discarded, the remaining layers are mixed, by which process about 2.5 - 3 ml of PRP is produced at the end with platelet concentration of 1.4 - 1.6 million/µl on average.

## Results

Sixty-four patients were selected for the study based on our inclusion criteria as mentioned above. Among these, 101 knees were given intra-articular injection (37 patients with bilateral knee injection and 27 patients with unilateral knee injection). Forty-seven knees were injected with only HA and 56 knees were injected with HA plus PRP.

**Fig. 1:**
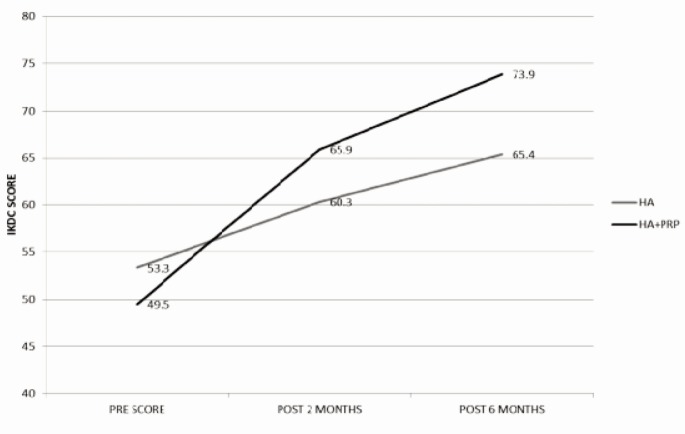
IKDC score during pre-injection, post 2 months injection, and post 6 months injection for HA and HA+PRP group.

As illustrated in Figure 1,both group of patients showed improvements in terms of functional status based on IKDC scores. Statistically significant improvements in IKDC scores were noted during two months post injection evaluation, with a mean value of 7.0 in patients receiving HA only and a mean value of 16.4 in patients receiving the combination of HA+PRP. The improvement was more apparent during the six months post injection evaluation whereby a mean value of 24.3 was obtained in patients who had received the combination of HA+PRP, in comparison with a mean value of 12.2 in patients who received HA alone as supported by statistical evidence in Table I.

**Table I:** Comparison between HA group and HA plus PRP group in improvement of IKDC score during the post 2 months and post 6 months evaluation

IKDC score	HA (SD)	HA + PRP (SD)	Mean Diff. (95% CI)	P Value
Improvement in IKDC score at 2 months post injection	7.0 (7.8)	16.3 (11.9)	-9.3(-13.2 – -5.4)	<0.05
Improvement in IKDC score at 6 months post injection	12.1(8.2)	24.3(13.7)	-12.1(-16.6 – -7.7)	<0.05

**Fig. 2:**
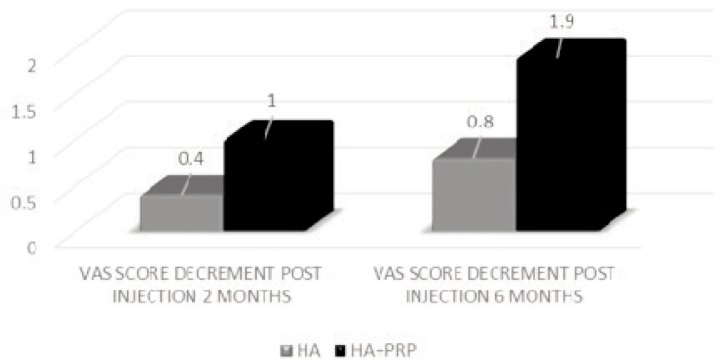
Improvement in pain severity demonstrated by the decrement in VAS score from baseline (pre-injection score) during 2 months and 6 months evaluation for both HA and HA+PRP groups.

In addition, progress was also evident via VAS scale in terms of decrement in the severity of pain in both the groups. It showed statistically significant – improved outcome in the long run as better pain control was achieved during the six months evaluation compared to two months evaluation in both groups. During the six months evaluation, the HA group demonstrated improvement with a mean score of 0.8. On the other hand, HA+PRP group showed better improvement in pain score with a mean score of 1.9 as demonstrated in Figure 2.

Further analysis which was done on variables such as gender, age and ethnicity was not statistically significant in terms of outcome.

## Discussion

The growing incidence of knee OA worldwide places an enormous economic burden with direct and indirect cost including medical management, rehabilitation, arthroplasty and loss of occupational productivity as a result of functional disability^1-3^. This study is mainly targeted to group of patients with grade III and IV knee OA in our centre who refused surgical intervention or were not fit for operation because of their underlying medical comorbidities. Hence, they resorted to analgesics as a temporary measure for pain relief and tend to be dependent on them in order to continue with their normal activities of daily living (ADL)^1,2^. Long term complications of dependency on analgesics are detrimental to one’s health^1,2^. It is envisaged that this study will make a significant contribution in improving the quality of life and pain control in these patients by providing HA or HA plus PRP as an optional treatment modality in these group of patients.

In our study, high molecular weight (HMW) HA was chosen instead of low molecular weight (LMW) HA mainly because the HMW HA (1.5x 10^6^ Daltons) closely resembles the molecular weight of endogenous HA (~2 x106 Daltons) in the extracellular matrix^27,28,30^. Studies have demonstrated that HMW HA down regulates the gene expression of osteoarthritis associated cytokines and enzymes in fibroblast like synoviocytes (FLS)^14,15,29^. In addition, HMW HA has anti-inflammatory property and regulates the suppressor T-cells for cell proliferation^29,30^. Supporting evidence also shows that the HMW HA typically resides longer in the synovial joint as compared to LMW HA^27, 30^. This property improves the efficacy of HMW HA in inhibiting glycosaminoglycan release from the articular cartilage and results in better outcome in the long run^28, 30^. This was evident in our study as more significant improvement was noted during six months evaluation compared to two months post-injection in both groups of patients as reflected in the IKDC and VAS scores.

A combination of HA and PRP was used in this study as newer experimental studies focus on promoting cartilage repair, whereby sheer attention has been directed toward autologous PRP^18-20,23,26^. PRP is a biological therapy with the goal of delivering concentrated platelets to accelerate healing and regeneration of articular cartilage^18,19^. The unique property of PRP that is enriched with essential growth factors induce differentiation of mesenchymal stem cells into chondrocytes and thereby increase cell proliferation^18,19,20^. Moreover, it also suppresses inflammatory mediators such as interleukin-1, encourages matrix deposition, and slow down degeneration^18-20^. Hence, growth factors help stabilize cartilage homeostasis and aid in articular cartilage repair^19,20^. An additional advantage of autologous venous blood versus synthetic chemicals is that it eliminates the risk of allergic reaction and possible transmission of infections^19, 20^. Recent studies have shown that the migratory ability of the proliferative cells is increased by the combination of plasma rich in growth factors with HA (+212% in comparison to growth factors alone, and +335% compared with HA alone)^23^. The enhancement of the migratory ability leads to better regenerating capability and slows down the natural progression of the disease^23^. Combination of HA and plasma rich in growth factors also halts the degeneration process and this positive biological interaction improves the efficacy of this intra-articular treatment, making it superior to analgesics alone which only provide temporary relief and do not treat the underlying pathology^23^.

Our study shows that combination of HA and PRP results in significant improvement in IKDC and VAS scores compared to HA injection alone. This implies that the addition of PRP with HA improves functional outcome and pain control for up to six months of duration. However, we could not comment further on synergism between the two compounds as we did not include a control group of patients who received PRP injections only.

Majority of patients in our study group were not active and were less mobile compared to the general population. The highest level of activities in 80% of our study population based on IKDC score were light activities such as walking, housework or yard work. Future studies would be on patients who were more active with greater demands being placed on their knees to determine whether the significant improvement in IKDC score can also be observed in such group of patients.

Being a retrospective study, our research have certain limitations such as inability to measure certain statistical values. Besides that, the short duration of the present study is one of the limitations. A longer duration follow up would provide more information on the long term functional outcome. Although the inclusion criteria for this study was in our consideration quite comprehensive, and there is still a possibility of selection bias since the study was based on treatment in a single institution.

## Conclusion

Our study is strongly suggestive that combination of HA and PRP to be more effective compared to HA alone. Hence, we suggest combination of intra-articular HA and PRP injection as an optional treatment modality in the treatment of grade III and IV knee osteoarthritis in terms of functional outcome and pain control for up to 6 months of duration when surgical treatment is not an option.
